# The European Medical Corps: first Public Health Team mission and future perspectives

**DOI:** 10.2807/1560-7917.ES.2017.22.37.30613

**Published:** 2017-09-14

**Authors:** Joana M Haussig, Ettore Severi, Jonathan HJ Baum, Veerle Vanlerberghe, Amparo Laiseca, Laurent Defrance, Cristina Brailescu, Denis Coulombier, Josep Jansa

**Affiliations:** 1 Postgraduate Training for Applied Epidemiology (PAE), Robert Koch Institute (RKI), Berlin, Germany; 2 European Programme for Intervention Epidemiology Training (EPIET), European Centre for Disease Prevention and Control (ECDC), Stockholm, Sweden; 3 European Centre for Disease Prevention and Control (ECDC), Stockholm, Sweden; 4 European Commission, Directorate-General for European Civil Protection and Humanitarian Aid Operations (DG-ECHO), Brussels, Belgium; 5 Mercator Fellowship, Berlin, Germany; 6 Institute of Tropical Medicine, Unit of General Epidemiology and Disease Control, Public Health Department, Antwerp, Belgium

**Keywords:** Angola, epidemiology, European Medical Corps, Outbreaks, public health, yellow fever, rapid response, emergency response

## Abstract

The 2013–2016 Ebola epidemic in West Africa challenged traditional international mechanisms for public health team mobilisation to control outbreaks. Consequently, in February 2016, the European Union (EU) launched the European Medical Corps (EMC), a mechanism developed in collaboration with the World Health Organization (WHO) to rapidly deploy teams and equipment in response to public health emergencies inside and outside the EU. Public Health Teams (PHTs), a component of the EMC, consist of experts in communicable disease prevention and control from participating countries and the European Centre for Disease Prevention and Control (ECDC), to support affected countries and WHO in risk assessment and outbreak response. The European Commission’s Directorate-General European Civil Protection and Humanitarian Aid Operations and Directorate-General Health and Food Safety, and ECDC, plan and support deployments. The first EMC-PHT deployment took place in May 2016, with a team sent to Angola for a yellow fever outbreak. The aims were to evaluate transmission risks to local populations and EU citizens in Angola, the risk of regional spread and importation into the EU, and to advise Angolan and EU authorities on control measures. International actors should gain awareness of the EMC, its response capacities and the means for requesting assistance.

## The European Medical Corps

The Ebola virus disease epidemic in West Africa in 2013–2016 revealed shortcomings in the organisation of the international response to public health emergencies, such as the lack of rapidly deployable medical and public health experts, in addition to logistic and management challenges. Traditionally, support to outbreak control relies on the rapid mobilisation of non-governmental organisations (NGOs) and experts to cover the public health dimension through the Global Outbreak Alert and Response Network (GOARN) mechanism. This mobilisation scheme has allowed the successful control of Ebola outbreaks, among others, including large outbreaks such as the one that occurred in Gulu, Uganda in 2000–2001 [1]. However, the scale of the 2013–2016 West Africa Ebola epidemic called for a much larger mobilisation than that readily achievable through NGOs and GOARN alone [2]. On 8 August 2014, under the International Health Regulations (IHR 2005) the World Health Organization (WHO) Director-General declared the Ebola epidemic in West Africa a Public Health Emergency of International Concern (PHEIC). Invoking the PHEIC, the WHO Director-General cited the need for a coordinated international response to mobilise resources to support affected countries in controlling the epidemic [3]. As a result, the European Centre for Disease Prevention and Control (ECDC) mobilised 89 experts for WHO, who were deployed as public health teams to support field operations, along with the mobilisation of resources through the Directorate-General for European Civil Protection and Humanitarian Aid Operations (DG ECHO) and the European mobile laboratories [4]. At the height of the Ebola crisis, Germany and France proposed the White Helmets initiative, establishing a reserve pool of health experts to be mobilised swiftly and deployed in areas suffering health emergencies [5]. In February 2016, to implement the lessons learned from the 2013–2016 Ebola outbreak and in accordance with the European Consensus on Humanitarian Aid [6] that sets the EU commitment to the fundamental principles of humanitarian aid and to the humanitarian imperative, the European Union (EU) launched the European Medical Corps (EMC), enabling EU and other countries participating in the EU Civil Protection Mechanism (EUCPM) to rapidly deploy teams and equipment in response to public health emergencies and disasters with health and humanitarian dimensions both inside and outside the EU. Here we describe the implementation of the EMC and the first deployment of an EMC Public Health Team (PHT) during the 2016 yellow fever outbreak in Angola. We also highlight how the new EU mechanism contributes to the rapidly evolving structures, such as the emergency medical team developed in WHO and in other countries, that respond efficiently to global public health threats and prevent humanitarian crises and health disasters.

### European Emergency Response Capacity and European public health teams

**Figure 1:**
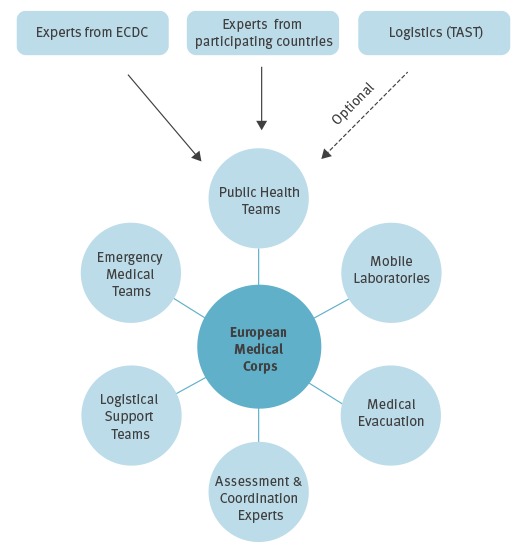
Response assets under the European Medical Corps

ECDC: European Centre for Disease Prevention and Control; TAST: Technical Assistance Support Team.

The EMC is part of the European Emergency Response Capacity, established under the EUCPM. The EMC includes emergency medical teams, public health teams, mobile laboratories, medical evacuation capacities and logistical support (Figure 1). The EMC’s comprehensive approach to dealing with medical and public health consequences of disasters also represents the EU’s contribution to the global health emergency workforce, and is therefore developed in close collaboration with WHO [7,8]. By August 2016, 11 participating countries had offered teams and equipment to the EMC.

European PHTs are one of the main components of the EMC and aim to provide teams of experts in prevention and control of communicable diseases to support activities in the affected country, under the overall coordination of the national authorities of the affected countries and WHO. PHTs must have the capacity to be deployed rapidly to assess public health risks and needs related to a health emergency, or to support response operations. PHTs are assembled ad hoc with public health experts from participating countries and ECDC. These teams can therefore serve to support WHO rapid response teams dealing with public health events of international concern. The mechanism to deploy PHTs follows the approach set up for deploying EUCPM teams, and gives the European Commission, including its various services that are interested in a specific mission, e.g. DG International Cooperation and Development (DEVCO) and DG Research and Innovation (RTD) and ECDC the role of shaping the mission and including competent public health bodies within participating countries in the planning of the mission. The mechanism is activated by a request for assistance by an EU country, a third country or a United Nations (UN) agency; the European Commission (EC) can also propose activating the mechanism to affected countries or to WHO. Whether or not the request for assistance comes from WHO, communication and coordination with WHO starts immediately. ECDC and the EC clarify the terms of reference for the mission.

 ECDC defines the expert profiles needed, e.g. clinical, epidemiological and microbiological expertise, language skills, knowledge of the mission setting, on which basis a request for experts for the mission is circulated to participating countries through the EC and ECDC networks. Participating countries propose experts for the mission; ECDC reviews and shortlists the proposed experts, the EC accepts and confirms the list of experts, and proposes them to the requesting authority, or WHO if the mission is a joint effort (for joint missions, WHO may also be involved in the process of expert selection). The European PHT is then deployed with the Emergency Response Coordination Centre (ERCC) of DG ECHO covering the logistical and financial needs of the mission. ECDC provides technical and scientific leadership during the preparations and during the mission.

A security briefing is organised by ERCC before departure; the experts deployed fall under the security rules of DG ECHO and are covered by special insurance while on mission, contracted by the EC. The EU delegation in the country of the deployment helps the PHT with on-the-spot logistical assistance and knowledge of the local health context. Additional support on health policies and context of the mission can be provided by regional DG ECHO staff. The PHT reports during its mission to the national authority, WHO or the organisation that requested the deployment, and also to the ERCC and ECDC. At the end of the mission the team has to deliver a technical report [9].

## The first European Medical Corps Public Health Team mission to assess the yellow fever epidemic in Angola

The Republic of Angola experienced an epidemic of yellow fever starting in December 2015. On 22 January 2016, the IHR focal point for Angola notified WHO of the ongoing yellow fever epidemic. During the epidemic, all 18 provinces of the country reported suspected cases with 14 provinces reporting also confirmed cases. By 30 April 2016 (when the EMC mission was approved), the Angolan Ministry of Health (MoH) had reported 2,023 cases and 258 deaths [10]. In response to the epidemic, a large-scale vaccination campaign was launched [11]. However, the vaccination efforts were hampered by lack of vaccine at the international level and by logistical constraints in Angola [12-14].

### Rationale for the mission

According to WHO estimates, 34 countries in Africa and 13 in Central and South America have conditions suitable for local (autochthonous) yellow fever transmission [15]. During the 2015–2016 epidemic in Angola the spread of confirmed cases of yellow fever from Angola to China, Kenya and the Democratic Republic of the Congo was documented, highlighting the potential for further international spread [14]. Although yellow fever has never been transmitted by the local *Aedes* species in south-east Asia, susceptible human populations and *Aedes aegypti* mosquito vectors are present, and an epidemic of yellow fever would have devastating consequences in the non-immune human population.

A risk assessment published by ECDC on 25 March 2016 considered the risk of importation of the virus into the European competent vector population through viraemic travellers to be limited [16]. However, it was noted that the level of evidence available from different sources reporting on this epidemic did not provide a robust basis for the risk assessment.

In view of the alarming situation in Angola, and considering that the competent vector *Aedes aegypti* is present in some areas of Europe and the EU (e.g. the island of Madeira, the Overseas Countries and Territories and Outermost Regions of the EU and the Black Sea region of Europe), the EC and ECDC saw added value in sending a team of public health experts to Angola. This mission was the first deployment of an EMC PHT and was organised with the support of the Government of Angola and in close collaboration with WHO, the EU Delegation in Angola and field partners.

## Mission objectives

The objectives of the mission were to advise authorities in Angola and in the EU on appropriate public health measures and to evaluate the risk to European citizens residing in or visiting Angola, the risk of further regional spread and of importation into areas of the EU where competent vectors are present. These objectives involved liaising with WHO and all those already deployed in the field to better understand the situation and review the information available concerning the epidemiological characteristics of the yellow fever epidemic in Angola, describing the characteristics of cases, at-risk groups, the dynamic of the epidemic, areas with local transmission and vaccine coverage.

## Organising the mission

Upon the proposal from the EU, the Government of the Republic of Angola requested an assessment mission, which was initiated by the EC and ECDC. The deployment of the expert team took place within the EUCPM framework. The ERCC organised the deployment of the team and ensured coordination with other EU services. ECDC provided scientific and technical leadership, providing terms of reference before the start of the mission and epidemiological support during the mission and the report preparation. The EC Directorate-General Health and Food Safety (DG SANTE) supported the mission, bringing experience in health policies for the prevention and control of health threats. The EU Delegation in Angola provided essential logistical support, background and contact information.

### Composition of the European public health team and coordination with other teams

**Figure 2:**
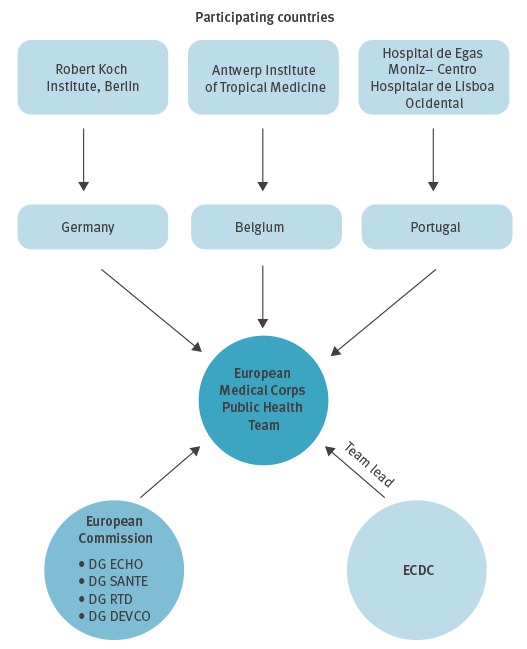
Institutions participating in the first European Medical Corps Public Health Team mission to Angola, May 2016

The team included experts from ECDC, DG ECHO and three EU countries (Germany, Belgium and Portugal) and consisted of a team leader (ECDC), two epidemiologists (ECDC and Robert Koch Institute, Postgraduate Training for Applied Epidemiology, Germany), a vector-borne disease epidemiologist expert (Antwerp Institute of Tropical Medicine, Belgium), a clinical expert (Hospital de Egas Moniz– Centro Hospitalar de Lisboa Ocidental, Portugal), a public health expert (DG ECHO, European Commission, Mercator Fellowship, Germany), an ERCC liaison officer (DG ECHO, European Commission) and a health expert based in the Democratic Republic of the Congo (DRC) (DG ECHO, European Commission) (Figure 2).

DG DEVCO: Directorate-General International Cooperation and Development; DG ECHO: Directorate-General European Civil Protection and Humanitarian Aid Operations; DG RTD: Directorate-General Research and Innovation; DG SANTE: Directorate-General Health and Food Safety; ECDC: European Centre for Disease Prevention and Control.

The mission objectives were addressed through meetings with senior public health officials from the MoH and the national public health directorate of Angola, clinicians from public hospitals in Angola, the WHO incident manager, the WHO country representative and other WHO officials, medical staff, entomologists and vector-control technicians from the Cuban Cooperation team (permanently based in Angola), epidemiologists from the United States (US) Centers for Disease Control and Prevention (CDC), experts from the Chinese Center for Disease Control and Prevention, medical and other staff from Médecins sans Frontières, and UN staff. The available epidemiological information was reviewed and discussed with the partners in the field.

**Figure 3:**
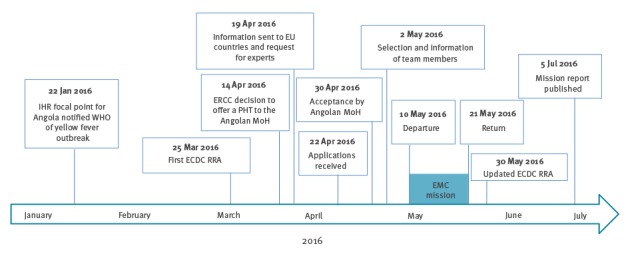
European Medical Corps Public Health Team mission to Angola timeline

ECDC: European Centre for Disease Prevention and Control; EMC: European Medical Corps; ERCC: Emergency Response Coordination Centre; EU: European Union; IHR: International Health Regulations; MoH: Ministry of Health; PHT: Public Health Team; RRA: Rapid Risk Assessment; WHO: World Health Organization.

Field visits in Luanda and two other provinces (Huambo and Huila) included visits to healthcare facilities (hospitals, primary care centres), provincial and municipal health authorities, public health activities (vaccination campaigns, risk communication, vector control) and the international airport of Luanda (Figure 3).

## Outputs of the mission

A technical mission report [12] was written and rapidly shared with DG ECHO, DG SANTE, the Angolan MoH and WHO, and made publicly available on the ECDC website. Furthermore, the findings from the mission were reflected in the first update of the ECDC rapid risk assessment on the outbreaks of yellow fever in Angola, DRC and Uganda [17].

The team concluded that at the time of the mission all regions in Angola should be considered as areas at high risk of transmission of yellow fever. Large urban areas and the Angolan northern provinces, including the exclave of Cabinda, represented a significant risk for international spread. The risk of importation in other countries, including the EU, could be successfully mitigated through the effective implementation of the recommendation of the IHR emergency committee on 19 May 2016 to screen at points of entry and exit for proof of yellow fever vaccination in the affected countries [18].

The mission report included recommendations on a wide field of topics such as surveillance, clinical management of suspected yellow fever cases, laboratory capacities, vaccination campaigns, vector-control activities and travel-related measures.

## Discussion and conclusion

In creating and deploying the PHT, the EU implemented one of the main lessons learned from the 2013–2016 Ebola epidemic, where public health expertise provided crucial support to the outbreak response, and complied with the European Consensus on Humanitarian Aid. The new mechanism demonstrated the ability to rapidly deploy public health experts selected for the objectives and context of the mission. Each team is selected from a pool of experts from participating countries with complementary professional profiles as well as language expertise fitting the needs of the deployment. DG ECHO’s ERCC is able to provide timely assistance during both the organisation and the deployment phase. In this regard, PHTs can be assembled to respond to a wide variety of scenarios and can be deployed at all stages of a health crisis, including supporting early-stage capacity building or needs assessment. PHTs can also provide guidance and advice to EU humanitarian and development actors, and enhance knowledge for EU internal preparedness purposes. These practices can maximise the benefits brought to the affected countries, limiting the usual challenges associated with an external intervention. The joint planning process between DG ECHO, DG SANTE and ECDC fosters coordination and cooperation at the EU level, including other EU services that may be involved if needed.

Ensuring that EU public health experts who may be sent on a PHT mission are sufficiently trained is an important challenge. The variety of outbreaks that may be faced by the PHT does not allow a standard training of the whole pool, but requires additional mechanisms for rapid training before deployment. To this end, and to ensure good understanding and coherence of PHT deployments, training a pool of selected team leaders may also be pursued in the future.

The role of EMC PHT is to work synergistically with WHO and the MoH of countries affected by a health emergency in assessment and response activities. Close collaboration with all partner organisations in the field is an essential principle in EMC’s involvement in these activities.

The experts deployed in the first EMC PHT mission were able to address the main mission objectives: to translate their field observations into recommendations for strengthening containment activities in Angola and to refine the assessment of the risk for importation in the EU.

The mission occurred 4 months after the declaration of the outbreak; future deployments of EMC PHT could occur at an earlier time point to allow for timely threat assessment and prompt implementation of response measures. These missions may need to be linked to follow-up actions by the EMC and partner organisations such as deployment of further experts based on the areas of need identified during the assessment mission.

The Angola mission was a relatively short assessment mission, and while the team cooperated with all stakeholders, it was not integrated within the response structure. For future response deployments, coordination structures and working arrangements have to be developed with WHO and GOARN, as well as the government and response actors in the affected country. Moreover, cooperation with other EMC assets like Emergency Medical Teams or mobile laboratories should be fostered. An option could be to embed public health experts within Emergency Medical Teams. PHTs should also participate in international preparedness training and exercises, and the scenarios for such activities should reflect possible health dimension of the event. Awareness of the EMC, its response capacities and the means for requesting assistance should be increased among international actors to ensure information exchange and timely activation.

The ad hoc assembled PHT is one concept in a fast developing field. Similarly to the EU, WHO and participating countries are undergoing reforms as a result of the Ebola crisis and developing new capacities [19-22]. PHTs, along with other EMC assets like the mobile laboratories and the medical evacuation capacities, are expected to provide GOARN, WHO rapid response teams and other partners with the invaluable opportunity to have highly flexible teams of qualified experts rapidly available for emergency deployment through one single easy-to-activate mechanism. It will be interesting to observe how these new mechanisms will evolve and influence the development and deployment of public health expertise in the future.
